# Cost-Minimized Nutritionally Adequate Food Baskets as Basis for Culturally Adapted Dietary Guidelines for Ethiopians

**DOI:** 10.3390/nu11092159

**Published:** 2019-09-09

**Authors:** Abdi Bekele Gurmu, Esa-Pekka A. Nykänen, Fikadu Reta Alemayehu, Aileen Robertson, Alexandr Parlesak

**Affiliations:** 1 Global Nutrition and Health, University College Copenhagen, 2200 Copenhagen, Denmark (A.B.G.) (E.-P.A.N.) (A.R.); 2 Academic Center of Excellence for Human Nutrition, Hawassa University, Hawassa P.O. Box 5, Ethiopia

**Keywords:** cost of diet, malnutrition, food baskets, linear programming, food accessibility

## Abstract

The high prevalence of undernutrition, especially stunting, in Ethiopia hampers the country’s economic productivity and national development. One of the obstacles to overcome undernutrition is the relatively high cost of food for low economic groups. In this study, linear programming was used to (i) identify urban and rural nutritionally adequate food baskets (FBs) with the highest affordability for an Ethiopian family of five and (ii) create urban and rural FBs, optimized for cultural acceptability, which are affordable for a family with the lowest income. Nutritionally adequate rural and urban FBs with highest affordability cost as little as Ethiopian Birr (ETB) 31 and 38 (~USD 1.07 and 1.31), respectively, but have poor dietary diversity (16 and 19 foods). FBs that cost ETB 71.2 (~USD 2.45) contained 64 and 48 foods, respectively, and were much more similar to the food supply pattern reported by FAO (15% and 19% average relative deviation per food category). The composed FBs, which are affordable for the greater part of the Ethiopian population, may serve as a basis for the development of culturally acceptable food-based dietary guidelines. These guidelines would recommend a diet composed of approximately up to 60% cereals, up to 20% roots and tubers, 10% legumes, and 10% fruits and vegetables by weight, plus only a small share from animal foods.

## 1. Introduction

Malnutrition remains a major public health problem in Ethiopia as 38% of children under the age of five are stunted and 24% are underweight. Moreover, 27% women and 37% of men are underweight [1]. This leads to high child morbidity and mortality and high susceptibility to both communicable and non-communicable diseases (NCDs) [1]. In addition, stunting is associated with the high cost of care for sick children, along with poor schooling and a lack of economic productivity [2,3].

Micronutrient deficiencies, including iron, vitamin A, and iodine, are of public health concern in Ethiopia. Eighteen percent of women in reproductive age, 34% pre-school children, and 7% school-aged children suffer from iron deficiency anaemia [4]. This prevalence is exacerbated by insufficient intake of folic acid and vitamin B12 [5,6]. Vitamin A deficiency, which is associated with night blindness and retinopathy, is present in 38% of the Ethiopian population [7]. Iodine deficiency has resulted in a goitre prevalence of 40% among children and 36% among women, i.e., in rural environments [4], with 39–70% of the population being at risk, particularly females of reproductive age [8,9].

There is also the emergence of a double burden of malnutrition as the prevalence of overweight and obesity is starting to rise, i.e., in the urban parts of the country. In 2011, the prevalence of overweight and obesity was 8% in women and 3% in men [10] and, five years later, (2016) this had increased to 28% in women and up to 16% for men [1]. Accordingly, hypertension, diabetes, and cardiovascular disorders are becoming an issue in Ethiopia [11]. Future Ethiopian health-promoting nutrition strategies, including dietary guidelines, are needed to prevent a worsening of this double burden of malnutrition [3]. It appears that no food-based dietary guidelines have been developed by the Ethiopian authorities [12].

Poverty and lack of knowledge are considered major obstacles to eating a nutritionally adequate diet, because foods rich in micronutrients, e.g., meat, may be too expensive [13,14,15].

Linear programming (LP) methodology has been useful for creating recommendations for affordable, locally available foods that provide all recommended nutrients while being culturally acceptable [13,16]. Linear programming requires access to (i) a database with the nutrient composition of foods, (ii) recommended nutrient and energy intakes, (iii) cost of foods, and (iv) the relevant cultural constraints [16,17,18,19].

The main goal of this study was to develop a basis for food-based dietary guidelines for Ethiopia that at the same time (i) uses linear programming, (ii) optimizes for similarity towards national food supply data to achieve cultural acceptability, and (iii) compares these data with a national food intake survey. To achieve this, daily food baskets (FBs) for an Ethiopian family of five were constructed that fulfill all Ethiopian recommended nutrient intakes (RNIs) at minimal cost while also being adapted for cultural acceptability. For this purpose, linear programming is used to maximize the similarity of the optimized FBs, which are based on locally available food. These food baskets can hopefully be used as a basis for developing food-based dietary guidelines (FBDGs) for Ethiopia.

## 2. Materials and Methods

### 2.1. Food Prices

Prices were collected from five urban marketplaces and supermarkets (for 205 foods) around the centre of Addis Ababa, plus prices from four marketplaces (for 190 foods) in rural areas close to Addis Ababa (Addis Ababa Zuriya) (Supplementary Table S1). One to five prices for each food item were collected at each supermarket or market place, and the median value used for all optimizations. All prices were collected in August and September 2016 with the help of local Ethiopians to reduce the likelihood of price increases for non-locals. The range of foods was limited to either raw or minimally processed commodities, such as yogurt, pasta, and cheese, to ensure food composition data were available and because the processing of foods is associated with increased cost (Supplementary Table S1) [16].

### 2.2. Nutrient Contents

The Ethiopian Food Composition Tables and West African Food Composition Database were primarily used to obtain nutrient values [20,21]. In the case of missing data, the following food composition tables and databases from the following countries were used: Mozambique [22], the United States (SR28) [23], the UK (CoFID) [24], Finland (Fineli) [25], or Norway (Matvaretabellen) [26]. Information on the non-edible proportion of foods such as skin, bones, fruit pips, etc. was taken from the same databases.

### 2.3. Energy Requirements and Recommended Nutrient Intakes

The daily estimated energy requirements (EER), the acceptable macronutrient distribution ranges (AMDR), and the upper and lower limits of recommended nutrient intakes (RNIs) for each family member were taken from FAO/WHO recommendations [27,28]. As the average household size in Ethiopia is 4.7 (3.8 in urban and 4.9 in rural areas) [29], all food baskets were calculated for a reference family of five. This family consisted of a woman aged between 30 and 59.9 years, with 65 kg of body weight; a 30 to 59.9 years old man with 70 kg of body weight, both having an assumed physical activity level of 1.75 [27]; two girls of whom one was 5 to 5.9 years old and the other aged 14 to 14.9 years old; and a boy 8 to 8.9 years old. The ages of the children were selected to cover a broad age range, and the adolescent in the family was chosen to be female to have at least two individuals in the family who have a high RNI value of iron. Table 1 provides information on the energy and nutrient recommendations applied as constraints during linear programming (Table 1).

Reference values on body weight were taken from the DHS report from 2014 [1] and from an analysis conducted by Subramanian and colleagues in 2011 [30].

### 2.4. Optimization of the Local Food Baskets Using Linear Programminge

Recommendations for health promotion, disease prevention, and nutrient intakes, along with cultural acceptability were optimized according to cost. Similar optimizations have been achieved using linear programming (LP) [13,16,31]. In LP, an algorithm is used to minimize or maximize a set of linear functions while applying specific constraints.

The LP algorithm uses three major components, which are a goal function, a set of decision variables, and a list of linear constraints [32]. The goal function was either (i) the sum over cost of each food or (ii) the sum of the non-negative values of the total relative deviation (TRD) of each food group. Food groups were defined based on those used in FAO’s most recent Food Balance Sheets (FBS) for Ethiopia in [33]. The TRD was calculated as:

(1) $$TRD=\sum_{i=1}^{N}\frac{\mathrm{abs}\left(m_{i}-M_{i}\right)}{M_{i}}$$

In Equation (1), *N* equals the number of food groups compared (here: *N* = 37) (Supplementary Table S1), *m_i_* is the weight of food in the *i*-th group of the food basket, and *M_i_* is the weight of food in the same group according to the FBS [13,19,33].

The decision variables were the weight of each food in the food basket. The constraints included the EERs and the recommendations on macro- and micronutrients, either as AMDRs or as RNIs (Table 1) [27,28]. The applied linear programming algorithm was the COIN Branch and Cut solver (CBC), which is a part of the OpenSolver add-in for Microsoft Excel, v. 2.9.0 [34].

Depending on where food prices were collected, two food baskets were calculated: (i) the Urban Food Basket (UFB), where prices were collected around central Addis Ababa (Shola, Piassa, Merkato, Kotebe and Alemgena and (ii) the Rural Food Basket (RFB), where prices were collected in rural areas (Chancho, Ijaji, Tullu Bollo, and Fiche).

The optimization was achieved in four steps:

- Food supply patterns, based on FBS, for a given energy constraint were assessed for cost and nutritional adequacy.
- Nutritionally adequate food baskets which met the EERs, AMDRs, and RNIs [27,28] were optimized for individual family members by cost. Thereafter, all foods from each family members’ food basket were summarized to give the whole family’s food basket according to affordability.
- When just optimized for cost alone, LP calculates only basic food baskets containing such few foods that they would be too monotonous and unacceptable over time [16,19]. Therefore, to increase cultural acceptability, the goal function was changed to minimize the TRD in relation to the EER-standardized weights of the FBS food groups [33]. The FBS comprised information on the specific nation-wide supply of 37 applicable foods and food categories in contrast to only 11 applicable food categories used in the latest available Ethiopian food consumption survey [4], and was therefore preferred as measure of food availability. The relative similarity of a food basket compared with the FBS is calculated as the average relative deviation (ARD) per food group (Equation (2)):
  (2) $$ARD=\frac{\mathrm{TRD}}{N}$$
- The weight of each food group in the FBs was standardized using the estimated daily energy recommendation of each family member (e.g., 2400 kcal/day for the mother) along with the daily per capita energy supply from the Ethiopian FBS (2284 kcal/day) [33]. For example, the FBS indicating a per capita supply of maize and products of 130.9 g/day was standardized: 130.9 g × 2400 kcal/2284 kcal = 137.6 g per day for the mother (Table 2). Increased dietary diversity was achieved by limiting the weight of each food within their specific group [19]. Therefore, the minimum number of foods per food group was either two (maximum contribution permitted of each food within its group was 80%) or three (maximum contribution permitted of each food within its group was 40%).

The maximum cost of a culturally adapted family FB was calculated for the lowest income group (corresponding to World Bank’s average income of the Ethiopian population below that of the 50th percentile). The upper cost limits set for the food basket were based on the daily cost of the households’ spenditure on food and beverages, adjusted for the yearly inflation rate [35]. Based on these figures, the maximum cost of a food basket for the lowest income was set at ETB 71.42 (~USD 2.45) per household/day.

Lastly, the optimized food baskets were compared with the most recently reported Ethiopian food consumption data, based on 24 h recall, which describe the intake of women, men, and children disaggregated into 11 food groups [4].

## 3. Results

### 3.1. Nutritional Analysis of the Food Balance Sheet Data

When standardized for energy intake recommendations for each family member, the food supply as reported by the FBS were lacking in: ω-6 PUFAs (only 46 to 59% of RNIs achieved), folate (only 50 to 79% of RNIs achieved), iodine (only 39 to 63% of RNIs achieved), and calcium (only between 28% and 53% of RNIs achieved) in both rural and urban areas. In addition, urban foods from the FBS data lacked vitamin C (only 36 to 54% of RNIs achieved) and rural foods lacked ω-3 fatty acids (only 54 to 55% achieved) and vitamin A for children (only 63 to 87% of RNIs achieved).

### 3.2. Least Costly Food Basket (FB) Meeting All Nutrient Recommendations

LP was applied to the food and price data collected, and the rural and urban food baskets with highest affordability, calculated for a family of five where all EERs, AMDRs, and RNIs are achieved, contained only 16 or 19 foods and cost 31.0 ETB or 38.1 ETB (~USD 1.07 and 1.31), respectively (Table 3). Both baskets contained mainly maize, seeds, vegetables, barley, beans, and other pulses, along with <1% (weight-%) beef liver, plus the urban basket contained milk products (7%) and the rural basket comprised eggs (2.4%). This composition makes the nutritionally adequate lowest-cost rural and urban FB differ considerably from the supply reported by the FBS (ARD values of 140% and 120%, respectively).

Those nutrients which were deficient in the food supply reported by the balance sheets are those mainly responsible for determining the cost of the least costly, but nutritionally adequate Ethiopian FB. In detail, these nutrients are omega-3 PUFAs, calcium, iodine, vitamins A, C, and E in the rural and urban FB along with folate in the rural basket. This means that as soon as these recommendations were achieved, the remaining nutrient recommendations were automatically fulfilled in this FB without additional cost, making them “active” constraints (Table 4). Additionally, iron (in the mother and the 14-year-old daughter), vitamin B12 (in the 14-year old daughter) and niacin (in all family members of the urban setting besides the father) constrain the cost of the FBs for the single family members and, consequently, that of the family FB. In both rural and urban food baskets, the upper limit recommendations for ω-6 PUFA, protein and sodium are active constraints for most family members (Table 4).

**Figure 1:**
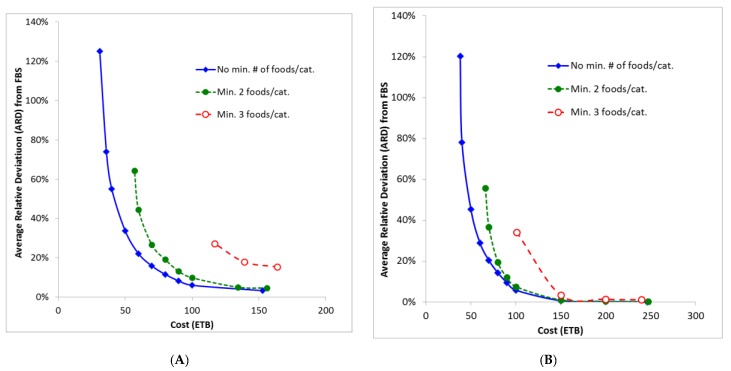
Average relative deviation (ARD) of the rural (Panel **A**) and urban (Panel **B**) food baskets compared with Food Balance Sheets (FBS) [33] depending on the maximum cost imposed; ETB: Ethiopian Birr.

The least costly food baskets showed low dietary diversity and also have the highest deviation from the reported food supply (FBS) (Figure 1).

### 3.3. Food Baskets Meeting All Nutrient Recommendations Plus Being Similar to FBS and with More Diversity

**Figure 2:**
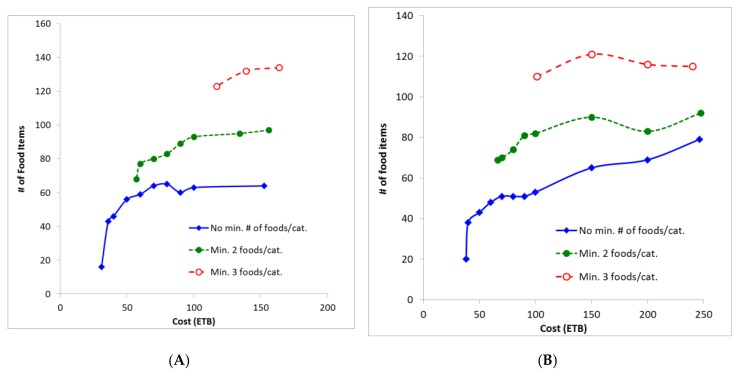
Number of foods contained in rural (Panel **A**) and urban (Panel **B**) food baskets depending on cost and minimum number of foods imposed per FBS food group [33]; ETB: Ethiopian Birr.

The food baskets, based on the food and price data collected, were optimized to be more similar to the Ethiopian FBS [33] by minimizing their total relative deviation (TRD). Those rural and urban food baskets, which were nutritionally adequate and most similar to the FBS (showing the lowest TRD value), contained 64 and 79 foods, and cost ETB 153 and 246 (~USD 5.26 and 8.46), respectively. When optimized for the lowest deviation from the FBS, the number of foods included in the rural FB did not increase beyond a cost of approximately ETB 80 (Figure 2).

The rural food basket that was most similar to the FBS showed an average relative deviation (ARD) of 3.3% compared with none (0%) in the urban one. Rural and urban baskets that were modeled to be less similar (i.e., deviated up to 10%) compared with the FBS, were less expensive and cost ETB 84 and 90 (~USD 2.89 and 3.1), respectively (Figure 1).

### 3.4. Food Baskets with Increased Dietary Diversity

When the LP model was constrained to contain a minimum of either two or three foods per food group, the rural FB contained 68–97 or 123–134 foods, respectively. Correspondingly, the urban FB contained 69 to 92 (minimum two foods/category) or 110 to 121 foods (minimum three foods/category) (Figure 2). With a minimum of two foods per food group, the cost raised to a minimum of ETB 57.0 or 66.1 (~USD 1.96 and 2.27) for the rural and urban FBs, respectively (Figure 2). At cost higher than ETB 66.1, enforcing at least two foods per food group increased diversity but did not increase the cost of the baskets compared with when no minimum number of foods per food group was enforced. For example, at a cost of ETB 100, the urban FB contained 53 different foods without diversification, 82 foods with a minimum of two foods per food group and 110 foods with a minimum of three foods per group (Figure 2, Panel B).

### 3.5. Baskets Calculated According to Household Spend and the Actual Nutrient Intake

As the lowest-income household has a maximum budget for food and non-alcoholic beverages of ETB 71.4, the rural and urban food baskets were constrained to this cost and were optimized for the least deviation (least TRD) from the FBS. These food baskets contained 64 and 48 foods in the rural and urban version, respectively, and their ARDs were 15.2% and 19.3%, respectively (Table 5 and Figure 1). The LP algorithm did not include the major part of foods being available at the markets as only 35% and 24% of the obtainable food items were included in the rural and urban food basket, respectively (Table 5 and Supplementary Table S1).

**Figure 3:**
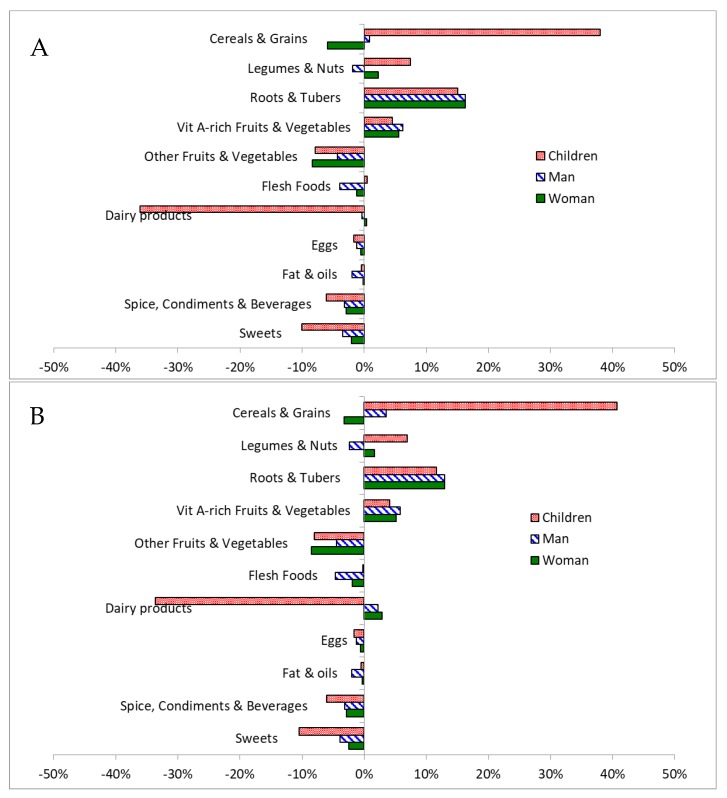
Relative differences between the nutritionally adequate rural (Panel **A**) and urban (panel **B**) FB for an Ethiopian family of five affordable for ETB 71.24 and the reported average consumption of eleven food categories in Ethiopia [4].

Constraining the cost to the level of what lowest-income families use for food and non-alcoholic beverages resulted in deterioration of dietary diversity through an increased share of (i) maize (at the expense of sorghum and teff) in the cereals & grains group, and (ii) legumes and nuts, along with a reduced share of dairy products, sweets, and roots and tubers (Table 5). These substitutions, particularly in relation to cereals and grains and dairy products, were most pronounced in the children’s FBs with respect to the EPHI’s nutritional intake survey [4] (Figure 3). The optimized FB affordable for ETB 71.2 differs for all family members in comparison to the reported food intake by increased amounts of roots and tubers and vitamin A-rich fruits and vegetables. At the same time, the recommended intakes of other fruits and vegetables, spice, condiments and beverages, and sweets are lower in the FB compared to the intake reported by EPHI’s nutritional intake survey.

## 4. Discussion

The results of the current study show that LP methodology can provide optimized food baskets for Ethiopia which are (i) nutritionally adequate, (ii) build on locally available foods, (iii) are affordable for a lowest-income family, and (iv) are, within the given cost constraint, optimised for highest possible similarity to national food supply patterns. The cost of such FBs with highest affordability in rural and urban areas was about 44% and 53% of the maximum cost assumed to be spent for food by a family with lowest income. These low-cost FBs, however, differed on average by more than 100% from the reported Ethiopian food group supply (FBS). Linear programming was also shown to be effective to minimize the average deviation from the reported food group supply to less than 20% when the FBs were allowed to cost the maximum of what can be afforded by a family with lowest income.

In 2016, more than one third (38%) of the children under five were stunted ([1]. Stunting is less common in Addis Ababa (15%), but more common in rural areas (up to 46%). Also, children from the poorest households and whose mothers have no education are more likely to be stunted (45% and 42% respectively) [1]. Stunting is an indication of chronic undernutrition which leads to increased risk of NCDs in adulthood, along with poor socio-economic development [3,36]. Stunting can be largely prevented if the family food eaten meets nutrient and dietary diversity recommendations.

### 4.1. Food Balance Sheet Data

Diet diversity in Ethiopia is in general extremely low, and while some regions have slightly higher dietary diversity than others, the limited diversity and overdependence on starchy staples is a nationwide problem in both rural and urban populations. Ethiopia scores the lowest of 125 countries according to the food quality dimension of the Oxfam “Good Enough to Eat” index, which measures diet diversification [37]. The results of this study show that the list of foods reported in the Ethiopian food supply (Table 1) [33] is deficient in PUFAs, iron, folate, iodine, calcium, and in vitamins A and C. These results are consistent with data from the Ethiopian food consumption survey [4] where only just over one third (38%) of children aged 6–23 months consumed foods rich in vitamin A and less than one quarter (22%) consumed foods rich in iron the day before the survey was conducted.

A more than six-fold regional variation exists between intakes of vitamin A rich foods in rural areas (11%) compared urban ones, e.g., Addis Ababa (69%) [4]. The intake of both vitamin A and iron-rich foods increase with household wealth and maternal education so that the prevalence of anaemia is more common in poorest households (68%), where women have no education (58%); and in rural (58%) compared with urban areas (49%) [1].

Most of the iron in the Ethiopian food supply (Table 1) comes from plant food sources which have low iron bioavailability [38]. Bioavailability can be improved by parallel consumption of meat or foods rich in vitamin C [39]. Therefore, an important recommendation within Ethiopian dietary guidelines would be to ensure that foods rich in vitamin C, such as citrus fruits, mango, papaya, and watermelon, are consumed at the same time as cereals in order to improve iron bioavailability.

According to the analyzed FBS data, iodine in Ethiopian food supply (Table 1) is insufficient. In contrast, the EDHS reported in 2016 that iodized salt is widespread and there are no large differences in quantities consumed by household wealth or residence [1]. Nine in ten families have iodized salt in their household and its use has greatly improved between 2011 and 2016 (15 to 89% households). However the concentration levels may be inadequate given the high number of women (36%) and children (40%) suffering from iodine deficiency [4] and optimum iodization of salt should be considered important in Ethiopia’s dietary guidelines.

### 4.2. Low Cost Food Baskets with Little Diversity

Given that the risk of chronic malnutrition is high in low income families [15], this study attempts to find the most cost effective nutritionally adequate food baskets (Table 3). Using the LP methodology with minimized cost as the goal function, a list of foods (16 and 19, respectively, for rural and urban) that fulfilled all nutritional recommendations for a family of five was calculated (Table 3), costing 31.0 and 38.1 ETB per day, respectively. These food baskets contained around (i) 58% cereals and grains, (ii) 18% roots and tubers, (iii) 10% legumes, (iv) about 8% of vitamin A-rich fruits, and (v) vegetables, plus the urban basket contained 4% dairy products.

However, these least costly nutritionally adequate food baskets (Table 3), being calculated for one day, contain only a low number of foods and thus a lack of dietary diversity. Moreover, there is a high deviation from the reported Ethiopian food supply (FBS) [33]. Thus, these baskets are unlikely to be acceptable in the long term. In order to design baskets that are more acceptable, it has been suggested to calculate examples where the foods selected are as similar as possible to either national dietary intakes or national FBS [13,19]. When local eating preferences are applied from the start as constraints into LP, it might not be possible to meet all RNIs by 100%. In a study aiming to develop a food guide for Benin that included local eating habits into the LP model, the lower thresholds of calcium, zinc, iron, and folic acid had to be reduced to 64–70% of the RNI recommended by the World Health Organization [40]. In contrast to this approach, the current paper suggests to improve a nutritionally adequate FB with highest affordability (Table 3), which is very distinct from prevailing food supply patterns, towards a FB that is as similar as possible to the FBS profile within the given maximum cost (Table 5).

### 4.3. Similarity to Food Balance Sheets

When baskets were optimized to mirror as closely as possible the food balance sheets, the number of foods increased to 64 and 79 and to an ETB cost of 153 and 246 per day for rural and urban baskets, respectively. Clearly, this cost is prohibitive for the majority of low income families. Even with a calculated average deviation from the food balance sheets of around 10%, the cost is still high at ETB 84 and 90 per day, respectively.

In this study, the optimized baskets discussed so far are either prohibitively costly or, if affordable, they are lacking in dietary diversity and so, in the long term, chronic malnutrition will develop. This is in line with results from the Ethiopian food intake survey [4] where children and women are reported to have insufficient intakes of several micronutrients. The authors recommend improved dietary diversity, including increased consumption of animal products, legumes, fruits, and vegetables. Moreover, they suggest increasing intakes of protein, vitamin A, vitamin C, zinc, iron, calcium, and folate. Indeed, according to WHO the minimum dietary diversity recommended for children is that their intake should come from at least four out of seven food groups [1,41] containing at least one fruit or vegetable in addition to cereals [41].

As shown in Figure 2, the cost-neutral steep increase of food items in the FBs is first effective at a cost around 70 ETB in both FBs. Thus, families not earning enough to cover this (i.e., those spending a maximum of 71.4 ETB) required further consideration in this study.

Given that 50% of Ethiopians have only on average ETB 71.4 per household/day to spend on food and non-alcoholic beverages, nutritionally adequate food baskets were optimized using a cost constraint corresponding to this amount. A list of 64 and 48 foods were generated for the rural and urban baskets, respectively (Table 5). The World Health Organization recommends, within the time frame of a week, a minimum of 20 to 30 biologically distinct food types, with an emphasis on plant foods, as a basis for dietary guidelines [42]. This WHO recommendation, even for Ethiopian households living on the lowest income, is fulfilled (Table 5). However, the deviation from the FBS is high (ARD values of 15% and 19%), indicating that the list of foods generated for these lowest income Ethiopians is not similar to their available food supply.

Compared with the most affordable nutritionally adequate baskets (rural: ETB 31, urban 38.1), the FBs affordable for ETB 71.4 contained (i) less legumes and nuts (10% vs. 18% and 15%, rural and urban, resp.), (ii) more roots and tubers (18% and 15% vs. none), (iii) similar proportions of vitamin A-rich fruits and vegetables (8% vs. 11% in both baskets), and (iv) less grains and cereals (58% and 61% vs. 67% in both FBs). Additionally, the urban basket contained more dairy products (4% vs. none) (Table 3 and Table 5). Thus, when the ETB amount used as cost constraint is higher, the LP optimization algorithm calculates a more diverse range of foods that matches more closely food availability and food intake (Figure 1) [33].

Clearly there is a large discrepancy between the amounts of ETB Ethiopian families have to spend compared with the cost of a nutritionally adequate, sufficiently diverse diet. For example, over half the vegetables and fruits are bought in local markets at relatively high prices [12]. Possibly as a consequence, none of the nutritionally optimized FBs contain anywhere near the WHO’s dietary guideline of at least 400 g of fruits and vegetables per day [42]. Indeed, Ethiopia scores lowest among 187 countries for fruit consumption, and second lowest for vegetable consumption [43]; for example, only 14% of women report consuming dark leafy vegetables on the previous day, only 10% any other vitamin A–rich vegetable or fruit, and only 18% other vegetables [4].

It is not clear why this intake is so low, and through Ethiopia’s Productive Safety Net Program [44], health and agricultural extension workers are promoting homestead gardens which, if adopted at scale, could improve the affordability of vegetables and fruit. Indeed, many rural families in Ethiopia produce their own food and therefore may be able to provide parts of the recommended FBs at lower cost, i.e., when focusing on foods that can provide micronutrients that are critical for the cost of the FB such as fruits and vegetables. A higher availability of fruits and vegetables would lead to falling prices, making these foods more readily available for everyone.

In the short term, it is practically unlikely that Ethiopians can implement a dietary guideline of at least 400 g of vegetable and fruit per day. This may exacerbate the risk of double burden of malnutrition where increasing rates of obesity, combined with the high prevalence of stunting [45] will lead to more NCDs. Indeed the prevalence of diabetes is estimated to double between 2011 and 2030, from 1.4 million to 2.7 million in Ethiopia [46]. To help prevent this escalation in NCDs, Ethiopian dietary guidelines are needed. The use of LP can help develop, especially for low income families, dietary guidelines and household practices that meet micronutrient and fibre recommendations while being as close as possible to the availability and reported intake of food in Ethiopia.

### 4.4. Similarity to the Ethiopian Food Consumption Survey

The amount of food groups in the FB optimized for the lowest income family differ considerably from the reported intakes (Figure 3) [4]. Given the cost of local foods, these differences can be explained by the circumstance that nutrients from cereals; legumes and nuts; vitamin A-rich vegetables; and roots and tubers are more affordable than those from dairy products; spices, condiments and beverages; and sweets. EPHI’s nutritional intake survey does not segregate the food intake data by family income, so it’s not known whether indeed these differences prevail in families with lowest income. However, it can be assumed that the differences between the optimized FB and the nutritional intake survey are a direct effect of the cost pressure as comparable differences occur in comparison to the supply reported in the FBS, i.e., a massive reduction in dairy products and sweets, and an increase in pulses and seeds [33].

### 4.5. Limitations

The cost of the food baskets in this study only applies to the purchase of food, and it does not cover expenses related to food preparation such as energy, transport, cooking equipment, and time. The FBs are designed for a reference Ethiopian family of five and do not apply to people with special nutritional needs such as pregnant women, older people, and people with food intolerance or allergy.

As indicated previously, food balance sheets only provide per capita estimates and do not contain information necessary to tailor individual solutions segregated by sex, age, and socio-economic status [47]. Also, FBS may lead to overestimation of food consumption and nutrient intake as they do not take into account avoidable food waste. However, unavoidable food waste has been considered by using yield information for each commodity, and previous studies have shown a close correlation between FBS data and food consumption estimates [47].

Ethiopia is a large country and food prices and availability may vary beyond the data used in this study. Additionally, the collected food prices may vary depending on the season. More than half of Ethiopians are Orthodox Christians [1] who practice fasting, which can last over two thirds of one year (250 days) [48], when they do not consume animal products such as meat or dairy. Therefore, more studies are needed to investigate the extent of veganism and religious fasting and its impact on malnutrition. Investigations, such as those proposed by Ethiopian Public Health Institute and Wageningen University in the Netherlands [49], are needed to look into what kind of Food Based Dietary Guidelines are needed and how they can be disseminated the best in areas where nutrition deficiencies are most prevalent. Such studies should also include home-grown food originating from, e.g., home gardens, which was not done in this study.

## 5. Conclusions

The nutritionally adequate family food baskets calculated using LP at the lowest possible cost differ significantly from Ethiopia’s food balance sheets. The lowest-cost rural and urban baskets cost ETB 31.0 and 38.1, contain only 16 or 19 foods, respectively, and so are very low in diet diversity and would not be sustainable in the long-term. Fifty percent of Ethiopians with the lowest budget for food and beverages (ETB 71.4 per household/day) therefore need support via homestead gardening or other ways to reduce the impact on the likely double burden of malnutrition. However, using LP, food baskets for families with ETB 71.4 per household/day to spend on food may serve as basis for dietary guidelines that can be followed by the majority of the Ethiopian people living in urban or rural areas close to large cities. The food supply for low-income families should consist of weight approximate to (i) 60% cereals, (ii) 15–20% of roots and tubers, (iii) 10% of pulses and nuts, (iv) 10% of vitamin A-rich fruits and vegetables, and (v) 5% milk or milk products. Cereals and grains can serve as the main source of energy while pulses provide protein. Bell pepper, cabbage, spinach, tomato and papaya supply vitamin A while roots and tubers provide minerals. Vegetable oils provide PUFAs and very small amounts of meat, especially liver and kidney, helping to satisfy iron and B12 recommendations. Future Ethiopian dietary guidelines should include recommendations that ensure ω-3 PUFAs, calcium, iodine, and vitamins A, C, and E from affordable sources are promoted. Future investigations could examine the feasibility and acceptability of these food baskets as a basis for the development of Ethiopia’s low income food based dietary guidelines. Given the size of Ethiopia, and the local food production and cultural diversity, future research should cover more areas of the country and possibly include home production of food.

## Figures and Tables

**Table 1:** Energy and nutrient intake recommendations applied during linear programming. The estimated energy requirement (EER) reference value is based on a physical activity level of 1.75 for both woman and man. The nutrient contents of all calculated food baskets lie within the indicated ranges of AMDRs and RNIs [27,28].

	Woman (30–59.9 Years)	Man (30–59.9 Years)	Boy (8 Years)	Girl 1 (5 Years)	Girl 2 (14 Years)
Energy (kcal)	2400	2950	1830	1330	2450
Protein (g)	54–90	58–110	26–69	16–50	41–92
Fat (g)	53–80	49–98	31–61	22–44	54–82
SFAs (g)	<26.7	<32.8	<20.3	<14.8	<27.2
PUFAs (g)	16–27	20–33	12–20	8.9–15	16–27
ω-3 PUFAs (g)	2.7–5.3	3.3–6.6	2.0–4.1	1.5–3.0	2.7–5.4
ω-6 PUFAs (g)	13–21	16–26	10–16	7.4–12	14–22
TFAs (g)	<2.7	<3.3	<2.0	<1.5	<2.7
Cholesterol (mg)	<300	<300	<300	<300	<300
Carbohydrates (g)	330–450	406–553	252–343	183–249	337–459
Fibre (g)	≥25.0	≥25.0	≥15.25	≥13.0	≥24.0
Total sugars (g)	<30.0	<36.9	<22.9	<16.6	<30.6
Na (mg)	<2000	<2000	<2000	<2000	<2000
K(mg)	≥3510	≥3510	≥2106	≥1831	≥3371
Ca (mg)	≥1000	≥1000	≥700	≥600	≥1300
Mg (mg)	≥220	≥260	≥100	≥76	≥220
Fe (mg)	≥29.4	≥13.7	≥8.9	≥6.3	≥32.7
Zn (mg)	≥4.9	≥7.0	≥5.6	≥4.8	≥7.2
Se (µg)	≥26	≥34	≥21	≥22	≥26
Iodine (µg)	≥150	≥150	≥120	≥90	≥150
Vit A-RAE (µg)	≥500	≥600	≥500	≥450	≥600
Thiamine (mg)	≥1.1	≥1.2	≥0.9	≥0.6	≥1.1
Riboflavin (mg)	≥1.1	≥1.3	≥0.9	≥0.6	≥1.0
Vit B6 (mg)	≥1.3	≥1.3	≥1.0	≥0.6	≥1.2
Vit B12 (µg)	≥2.4	≥2.4	≥1.8	≥1.2	≥2.4
Vit C (mg)	≥45	≥45	≥35	≥30	≥40
Vit D (µg)	≥5.0	≥5.0	≥5.0	≥5.0	≥5.0
Vit E (mg)	≥7.5	≥10.0	≥7.0	≥5.0	≥7.5
Folate (µg)	≥400	≥400	≥300	≥200	≥400
Niacin (mg)	≥16.0	≥14.0	≥12.0	≥8.0	≥16.0

SFA, saturated fatty acids; PUFA, polyunsaturated fatty acids; TFAs, trans-fatty acids; vit, vitamin; RAE, retinol activity equivalents; minerals and trace elements are indicated by their chemical symbols.

**Table 2:** Food supply pattern of an Ethiopian reference family as reported in the Food Balance Sheets (FBS) by FAO [33], adjusted for the individual EER [27]. The listed food amounts are in g/day.

	FBS Food Group	Woman (30–59.9 Years)	Man (30–59.9 Years)	Boy (8 Years)	Girl 1 (5 Years)	Girl 2 (14 Years)
Cereals & Grains	Wheat and products	102.2	125.7	78.0	56.7	104.3
	Maize and products	137.6	169.2	104.9	76.3	140.4
	Millet and products	22.4	27.5	17.1	12.4	22.9
	Rice (milled equivalent)	7.4	9.0	5.6	4.1	7.5
	Sorghum and products	83.4	102.6	63.6	46.2	85.1
	Barley and products	50.3	61.8	38.4	27.9	51.3
	Cereals, other	86.7	106.6	66.1	48.1	88.5
Roots & Tubers	Sweet potatoes	47.1	57.9	35.9	26.1	48.1
	Potatoes and products	21.8	26.7	16.6	12.1	22.2
	Roots, other	195.8	240.7	149.3	108.5	199.8
Pulses & Nuts	Peas	11.6	14.3	8.9	6.4	11.8
	Beans	6.8	8.4	5.2	3.8	7.0
	Soybeans	1.5	1.8	1.1	0.8	1.5
	Groundnuts (shelled)	1.8	2.2	1.4	1.0	1.8
	Pulses, other and products	33.3	40.9	25.4	18.5	34.0
	Sesame seed	2.5	3.1	1.9	1.4	2.6
Vit. A-rich Fruits & Vegetables + Other Fruits & Vegetables	Tomatoes and products	1.1	1.4	0.8	0.6	1.1
	Oranges, Mandarins	1.7	2.1	1.3	0.9	1.7
	Grapefruit and products	1.1	1.4	0.9	0.6	1.2
	Onions	7.2	8.8	5.5	4.0	7.3
	Vegetables, other	50.6	62.2	38.6	28.0	51.6
	Apples and products	0.2	0.3	0.2	0.1	0.2
	Bananas	9.2	11.3	7.0	5.1	9.4
	Pineapples and products	0.3	0.3	0.2	0.1	0.3
	Dates	0.1	0.1	0.0	0.0	0.1
	Fruits, other	10.8	13.2	8.2	6.0	11.0
Flesh Foods	Mutton & goat meat	5.1	6.3	3.9	2.8	5.2
	Poultry meat	2.2	2.7	1.6	1.2	2.2
	Meat, other and Fish, other	4.8	5.9	3.7	2.7	4.9
Eggs	Eggs	1.2	1.4	0.9	0.7	1.2
Dairy Products	Milk–excluding Butter	144.4	177.5	110.1	80.0	147.3
	Butter, ghee	0.6	0.8	0.5	0.3	0.6
Fats & Oils	Soybean oil	0.1	0.1	0.0	0.0	0.1
	Oil crops oil, other	9.3	11.5	7.1	5.2	9.5
Sweets	Sugar (raw equivalent)	19.9	24.4	15.1	11.0	20.3

**Table 3:** Nutritionally adequate rural and urban food baskets for an Ethiopian family of five optimized for cost alone.

		RFB			UFB		
FBS Food Groups	Food Name	Weight of Raw Food (g)	RD (%)	Cost (ETB)	Weight of Raw Food (g)	RD (%)	Cost (ETB)
Wheat and products	Wheat, whole grains, raw				7.8	−98%	0.13
Maize and products	Maize, white, flour refined	1192	+123%	8.65	903	+69%	9.03
	Maize, white, on cob, roasted				36.0		0.32
	Maize, white, whole kernel, dried	133		0.84			
	Maize, yellow, flour of whole-grain	80		0.56			
Sorghum and products	Sorghum, whole grain, raw	6.7	−98%	0.06	209	−38%	3.45
Barley and products	Barley, black, whole grain	128	−45%	2.91	33.2	−84%	0.81
Beans	Broad beans, split	19.3	+268%	0.80	154	+458%	4.31
	Kidney bean, big Abbaa choma	96.3		1.49			
Groundnuts (shelled eq.)	Groundnut paste				3.8	−48%	0.14
Pulses, other and products	Linseed, dried	35.3	−42%	0.76		−52%	
	Linseed, roasted				7.7		0.32
	Niger seed, raw	12.4		0.31			
	Niger seed, dried	41.6		1.29	56.3		2.03
Sesame seed	Sesame seeds, whole, dried	90.3	+680%	4.15	105	+932%	2.94
Vegetables, other	Spinach, raw	98.6	+11%	1.63	26.2	+6%	0.52
	Pepper, sweet, red, raw				143		3.44
	Pepper, sweet, green, raw				33.7		0.77
	Peppers, chili, raw	159		2.55			
	Ginger, roots, dried				13.0		0.83
Meat, fish, other	Beef liver, raw	14.3	−35%	1.36	16.0	−17%	1.32
Eggs	Egg, small, chicken, raw	51.9	+859%	3.11			
Milk–excluding butter	Milk, cow, 1.5% fat				79.7	−77%	3.19
	Yoghurt, whole milk, plain				54.0		3.40
Oilcrops oil, other	Niger oil				11.8	−69%	0.64
Spices, other	Salt (Iodized)	22.2		0.53	22.1		0.49
	**SUM**	**2183 (16 foods)**		**31.01**	**1916 (19 foods)**		**38.09**

FBS, Food Balance Sheets; RFB, Rural Food Basket; UFB, Urban Food Basket; RD, Relative Deviation from food category supply as reported by FBS [33]; ETB, Ethiopian Birr.

**Table 4:** Nutrients constraining the food baskets from being less expensive (so-called “active” constraints).

		Macronutrients			Minerals + Trace Elements				Vitamins					
		ω-3 PUFA	ω-6 PUFA	Protein	Na	Ca	Iron	Iod.	Vit. A	Vit. B_12_	Vit. C	Vit. E	Folate	Niacin
**Lower limit**	Woman	U R				U R	R	U R	U R		U R	U R	R	U
	Man	U R				U R		U R	U R		U R	U R	R	
	Girl1	U R				U R		U R	U R		U R	U R	R	U
	Girl 2	U R				U R	U R	U R	R	U R	U R	U R		U
	Boy	U R				U R		U R	U R		U R	U R	R	U R
**Upper limit**	Woman		R	U R	U R									
	Man		U R	U R	U R									
	Girl1		U R	R										
	Girl 2		U R	U R	U R									
	Boy		U R											

R, rural FB; U, Urban FB; Na, sodium; Ca, calcium; Iod., iodine.

**Table 5:** The nutritionally adequate Ethiopian rural (RFB) and urban (UFB) food baskets for the lowest income households (maximum cost of ETB 71.24).

			RFB			UFB		
EPHI Food Groups	Food Items (FBS)	Food Name	Amount of Raw Food (g)	RD	Cost (ETB)	Amount of Raw Food (g)	RD	Cost (ETB)
Cereals & grains	Wheat and products	Bread/rolls, white	99	0%	1.69	140	0%	2.5
		Bread, wheat, white				25.6		0.4
		Wheat flour, white	225		4.06	93.7		2.1
		Wheat, whole grains, raw	145		1.92	153		2.5
	Maize and products	Maize, white, whole kernel, dried	38.9	+64%	0.24		+127%	
		Maize, white, flour refined	1001		7.25	1264		12.6
	Millet and products	Millet, whole grain, raw	103	0%	0.62	90	0%	0.79
	Rice (Milled equivalent)	Rice, white, raw	15.0	0%	0.32		0%	
		Rice, Brown, wholegrain, raw	18.8		0.35	29.7		0.3
	Sorghum and products	Sorghum, mixed, enjera	23.0	−88%	0.35		−88%	
		Sorghum, whole grain, raw	21.6		0.19	41		0.68
	Barley and products	Barley, black, whole grain	114	−16%	2.60	172	−8%	4.2
		Barley, black, whole grain, roasted	79.7		1.47	13.7		0.3
	Cereals, Other	Teff, red, flour	198	−50%	3.51			
Roots & tubers	Sweet potatoes	Sweet potato, yellow, raw	122	−29%	2.62			
	Potatoes and products	Potato, raw	131	0%	1.74	87.8	0%	1.1
		Potato, Irish				178	−6%	3.7
	Roots, Other	False banana, bulla	92.9	−55%	1.21		−70%	
		Tuber/Anch’oyte, raw	315		10.41	237		7.9
Legumes & nuts	Peas	Pea, field, split	6.5		0.16	46.9	0%	1.7
		Peas, raw	11.9	0%	0.36			
		Pea, field, flour	23.3		0.56			
		Pea, field, whole, dried	11.7		0.50			
	Beans	Broad beans, split	2.0	0%	0.08	27.6	0%	0.8
		Kidney bean, big Abbaa choma	29.4		0.46			
	Soyabeans	Soya bean, whole, flour	1.5	0%	0.05	5.9	0%	0.2
		Soya bean, split, raw	5.3		0.13			
	Groundnuts (Shelled eq.)	Groundnut paste	1.4	0%	0.07	7.3	0%	0.3
		Groundnut, shelled, dried, raw	6.9		0.40			
	Pulses, Other and products	Niger seed, dried	250		7.76	222	+65%	8.0
		Lentils, split	3.8	+66%	0.09			
	Sesame seed	Sesame seeds, whole, dried, raw	11.6	0%	0.53	10.2	0%	0.3
Vit. A-rich fruits & vegetables	Tomatoes and products	Tomato paste, conc.	5.1	0%	0.10	1.3	0%	0.1
		Tomato, raw				3.2		0.1
	Vegetables, Other	Spinach, raw	135		2.22	113	0%	2.3
		Cabbage, green, raw	37.8	0%	0.55			
		Peppers, chilli, raw	59.9		0.96			
		Pepper, sweet, green, raw				91.5		2.1
	Oranges, Mandarines	Orange, raw	7.7		0.16		0%	
		Tangerine, fresh				6.7		0.1
	Lemons, Limes and products	Lemon, raw	5.3		0.13			
		Juice, lemon, unsweetened				4.6		0.1
	Fruits, Other	Papaya, fruit, ripe, raw	17.0		0.41	43.4	0%	0.9
		Pomegranate, raw	13.3	0%	0.21			
		Mango, pale flesh, raw	8.3		0.13			
		Peach, fresh	10.8		0.19			
Other fruits & vegetables	Onions	Onion, white, raw	32.9	0%	0.38	28.9	0%	0.4
	Apples and products	Juice, apple, canned or bottled	0.5		0.02	0.9	0%	0.0
		Apple, with skin, raw	0.5	0%	0.02			
	Bananas	Banana, yellow flesh, raw	42.4	0%	2.46	37.2	0%	0.6
	Pineapples and products	Pineapple, pulp, raw	1.2	0%	0.03	1.1	0%	0.0
	Dates	Dates, dried	0.3	0%	0.01	0.3	0%	0.0
Flesh foods	Mutton & Goat Meat	Goat, meat, raw	23.6	0%	1.18			
	Poultry Meat	Chicken, flesh + skin, raw	8.6	0%	1.22			
		Chicken, liver, raw	1.4		0.25	8.7	0%	1.8
	Meat, Fish, Other	Beef liver, raw	5.9	0%	0.56	8.8	0%	0.7
		Lamb, kidney, raw	12.0		0.54	1.2		0.1
		Beef, ground, 10 % fat, raw	3.2		0.15			
		Beef, tripe, raw	1.0		0.10			
		Beef, kidney, raw				9.3		0.7
Eggs	Eggs	Egg, small, chicken, raw	5.4	0%	0.32	4.8	0%	0.5
Dairy products	Milk—w/o Butter	Milk, cow powder, whole	45.7	−93%	3.97			
		Milk, cow, 0.5 % fat				47.4	−78%	1.9
		Milk, cow, 1.5 % fat				31.7		1.3
		Yoghurt, whole milk, plain				47.7		3.0
Fats & oils	Butter, Ghee	Butter, cow’s milk (salted)	1.6		0.11	2.5	0%	0.3
		Butter, cow’s milk	1.3	0%	0.15			
	Soyabean Oil	Soya oil	0.3	0%	0.03	0.3	0%	0.0
	Oilcrops Oil, Other	Vegetable oil	42.8	−1%	1.11			
		Coconut oil				4.8	0%	0.3
		Corn oil				8.7		0.5
		Niger oil				24.2		1.3
Sweets	Sugar	Sugar, brown	39.1	−58%	1.29			
		Sugar				23.6	−71%	1.2
	Sweeteners, Other	Honey	0.9	0%	0.09	0.8	0%	0.1
Spices & condiments	Spices, Other	Coffee, powder	0.5	0%	0.02			
		Vinegar	3.8		0.13	3.8	0%	0.1
		Salt (Iodized)	21.8		0.52	21.0		0.5
		**Sums**	**3706 (64 foods)**		**71.24**	**3427 (48 foods)**		**71.24**

RD, Relative deviation from the FBS; ETB, Ethiopian Birr; EPHI, Ethiopian Public Health Institute [4].

## Supplementary Materials

The following are available online at https://www.mdpi.com/2072-6643/11/9/2159/s1. Table S1: Foods and food groups included in the optimisation.

## Abbreviations

AMDRs	Acceptable Macronutrient Distribution Ranges
ARD	Average Relative Deviation
EER	Estimated Energy Requirement
EPHI	Ethiopian Public Health Institute
ETB	Ethiopian Birr
FAO	Food and Agriculture Organization of the United Nations
FBDGs	Food-Based Dietary Guidelines
FB	Food Basket
FBS	Food Balance Sheets
LP	Linear Programming
MUFA	Monounsaturated Fatty Acids
NCDs	Noncommunicable Diseases
PUFA	Polyunsaturated Fatty Acids
RD	Relative Deviation
RFB	Rural Food Basket
RNI	Recommended Nutrient Intake
TRD	Total Relative Deviation
UFB	Urban Food Basket
WHO	World Health Organization
