# Supplementary material for: Cost-Minimized Nutritionally Adequate Food Baskets as Basis for Culturally Adapted Dietary Guidelines for Ethiopians
Source: Nutrients. 2019 Sep 9;11(9):2159. doi: 10.3390/nu11092159 (PMC6770709; doi:10.3390/nu11092159)
Supplement: Supplementary file 1 [file nutrients-11-02159-s001.pdf]

nutrients

Gurmu et al.: Cost-Minimized Nutritionally Adequate Food Baskets as Basis for Culturally Adapted Dietary Guidelines for Ethiopians

Supplementary Table S1. Applied FAO Food Balance Sheet & EPI Categories and food groups [4].

FAO Food Balance Sheet Categories	Ethiopian Public Health Institute Categories	FAO Food Balance Sheet Food Groups	Examples of Foods
Cereals, Grains and their products	Cereals/Grains	Wheat and products	Baguette
		Maize and products	Maize, yellow, whole kernel, dried
		Millet and products	Millet, black, enjera
		Rice (Milled Equivalent)	Rice, Brown, wholegrain, raw
		Sorghum and products	Sorghum, mixed, enjera
		Barley and products	Barley, white, flour
		Cereals, Other	Teff, red, enjera
Starchy roots, tubers and their products	Roots and Tubers	Sweet potatoes	Potato, boiled
		Potatoes and products	Potato, raw
		Roots, Other	Taro, tuber, raw
		Yams	Tuber/Anch'oyte, raw
Legumes and their products	Legumes and Nuts	Peas	Peas, raw
		Beans	Broad beans, split
		Soyabeans	Soya bean, split, raw
		Pulses, Other and products	Groundnut paste
Nuts, seeds and their products		Groundnuts (shelled)	Lentils, split
		Sesame seed	Sesame seed, white, dried
		Nuts and products	Safflower seed, raw
Vegetables and their products + Fruits and their products	Vit A rich Fruits and Vegetables	Tomatoes and products	Tomato, red, ripe, raw
		Oranges, Mandarins	Orange, raw
		Grapefruit and products	Grapefruit, pulp, raw
		Lemons, Limes and products	Lemon, raw
		Citrus, Other	Juice, lemon, unsweetened
		Onions	Onion, white, raw
	Other Fruits and Vegetables	Vegetables, Other	Cabbage, white, raw
		Apples and products	Apple, with skin, raw
		Bananas	Banana, yellow flesh, raw
		Dates	Pineapple, pulp, raw
		Pineapples and products	Dates, dried
		Fruits, Other	Pomegranate, raw
Meat and poultry and their products	Flesh Foods	Mutton & Goat Meat	Goat, meat, raw
		Poultry Meat	Chicken giblets, raw
		Meat, Fish, Other	Beef liver, raw
	Eggs	Eggs	Egg, big, chicken, raw
Milk and its products	Dairy Products	Milk - Excluding Butter	Milk, cow powder, whole
Fat and oils	Fat and oils	Butter, Ghee	Butter, from cow's milk (without salt)
		Soyabean Oil	Soya oil
		Oilcrops Oil, Other	Vegetable oil
Sugar Crops and Sweeteners	Sweets	Sugar (Raw Equivalent)	Sugar
		Sweeteners, Other	Honey
Miscellaneous	Spices and Condiments	Spices, Other	Vinegar
